# Gender Modifies the Effects of Education and Income on Sleep Quality of the Patients with Coronary Artery Disease

**Published:** 2013-12-01

**Authors:** Shervin Assari, Maryam Moghani Lankarani, Davoud Kazemi Saleh, Khodabakhsh Ahmadi

**Affiliations:** 1 Department of Health Behavior and Health Education, School of Public Health, University of Michigan, Ann Arbor, Michigan, USA; 2 Center for Research on Ethnicity, Culture, and Health (CRECH), School of PublicHealth, University of Michigan, Ann Arbor, Michigan, USA; 3 Medicine and Health Promotion Institute, Tehran, IR Iran; 4 Universal Network for Health Information Dissemination and Exchange (UNHIDE), Tehran, IR Iran; 5 Clinical Research Unit, Baqiyatallah University of Medical Sciences, Tehran, IR Iran; 6 Baqiyatallah University of Medical Sciences, Tehran, IR Iran; 7 Behavioral Sciences Research Center, Baqiyatallah University of Medical Sciences, Tehran, IR Iran

**Keywords:** Gender, Sex, Education Status, Income, Coronary Artery Disease

## Abstract

**Background:**

This study aimed to investigate the interaction between gender and other socio-economic characteristics on sleep quality of the patients with Coronary Artery Disease (CAD).

**Methods:**

This cross sectional study was conducted on 717 patients with CAD. The socio- economic status (education level, income, marital status, and place of residence) was considered as the independent variable. Besides, the study outcome was the quality of sleep which was measured using Pittsburgh Sleep Quality Index (PSQI). Gender was considered as a possible effect modifier. Two-way ANOVA was used to evaluate the interaction between gender and socio-economic factors on sleep quality. As defined by Baron and Kenny, moderator was defined as a variable that affected the direction or magnitude of the association of interest.

**Results:**

Female gender, low education level, and low income were predictive of poor sleep quality. Among female (10.0 ± 4.3 vs. 7.6 ± 5.0, P < 0.05), but not male patients (6.7 ± 4.2 vs. 7.0 ± 4.2, P > 0.05), low education was associated with poor sleep quality. Also, among female (10.0 ± 4.3 vs. 5.7 ± 2.5, P < 0.05), but not male patients (7.0 ± 4.2 vs. 6.0 ± 3.8, P > 0.05), low income was predictive of poor sleep quality. Gender did not modify the effect of other socio-economic factors on sleep quality.

**Conclusions:**

Among female but not male patients with CAD, low education and income were associated with poor sleep quality. This information helps us better understand the mechanisms behind the poor sleep quality of the female patients with CAD. This is important because poor sleep is a prognostic factor among the CAD patients.

## 1. Background

Sleep quality is an essential component of well-being (1). Sleep quality is associated with the quality of life (2, 3), affects mortality (4, 5), and influences the immune function (6-8). Studying sleep quality in the context of Coronary Artery Disease (CAD) is essential because poor sleep increases the risk of cardiac outcomes among both the patients with CAD (9, 10) and the general population (11).

Quality of sleep contributes to development or progression of CHD (9-11). In fact, a growing body of evidence suggests that poor sleep increases inflammation that may contribute to undesired cardiovascular consequences (12). Different studies have shown that gender may change the link between sleep quality and inflammatory biomarkers (13-16). In one study, sleep quality predicted 5-year changes in inflammatory biomarkers in women but not men (12).

Numerous studies have focused on the effect of gender on sleep quality. Most of the literature has shown that sleep-related complaints (17), sleep-onset latency and awakenings (18), sleep satisfaction (19), and sleep disorders (20-26) are more common among women than men. There are, however, a few studies suggesting no effect of gender on sleep quality (19, 27, 28), or a better sleep quality among men (29, 30). Among the patients with CAD, as well, women are known to report poorer sleep quality and efficiency, longer sleep onset latency, and more difficulty in falling asleep (31, 32).

The studies that have compared the sleep quality of men and women have had a biological or social perspective. For instance, there are studies which have suggested that hormonal fluctuations may explain the sex differences in sleep quality (20). Also, there are recent studies that have questioned the biological mechanisms for lower sleep quality of women (33). The next approach focuses on the social factors that explain gender differences in sleep quality. For instance, research has suggested that gender differences in mental health may explain why women experience worse sleep (27). Although socio-economic characteristics also influence sleep quality, very few studies have investigated how such factors contribute to the gender differences in sleep quality (34).

Based on the Theory of Gender and Power, which was originally developed by Connell in 1987, the sexual division of labor, the sexual division of power, and the structure of cathexis are the main structures that shape gendered relationships in a society. Based on this theory, major gender differences in employment, income, and education result in power imbalances and subordination of women in the society. Due to such inequalities, women experience different life experiences that ultimately influence their health and well-being (35).

In this study, we explored gender differences in the quality of sleep of the patients with CAD from a social perspective. Based on the Theory of Gender and Power (35), we hypothesized that women with CAD may be more vulnerable to the effects of socio-economic factors on sleep quality compared to the men with CAD. To test our hypothesis, we investigated the association between low socio-economic status and sleep quality among the women and men with CAD.

## 2. Patients and Methods

In a cross-sectional design, 717 CAD patients were selected from an outpatient cardiology clinic in Tehran during 2006. The patients were selected through systematic sampling. The inclusion criterion was diagnosis of CAD by a cardiologist according to the clinical findings and angiography. On the other hand, having some family or occupational conditions, such as changing work hours, shift-work, or living with a roommate or spouse with shift-work, which intruded on proper sleep were considered as the exclusion criteria. The study protocol was approved by the Institutional Review Board of the university. Written informed consents were also obtained from all the patients.

Socio-economic data, including gender, age, self-reported family income, marital status, educational level, and place of residence (urban area vs. rural area) were registered. In addition, clinical data, such as angiographic findings, disease duration, and history of previous myocardial infarction, hypertension, and hyperlipidemia, and risk factors such as cigarette smoking and Body Mass Index (BMI), were recorded from the patients’ charts.

Pittsburgh Sleep Quality Index (PSQI) was used to evaluate the quality of sleep. PSQI measures self-rated sleep quality over the past month in the following seven areas: 1) subjective sleep quality (self- perception of the overall sleep quality), 2) sleep latency, 3) sleep duration, 4) habitual sleep efficiency, 5) sleep disturbances, 6) use of sleeping medication, and 7) daytime dysfunction (problems experienced during the day owing to disordered sleep). Each component is scaled from 0 to 3, with 0 and 1 - 3 representing normal and abnormal conditions, respectively. The sum of scores for these seven components yields a total score which ranges from 0 - 21. This questionnaire has a sensitivity of 89.6% and a specificity of 86.5% in distinguishing good and poor sleepers (36). PSQI has been frequently used for Iranian populations (37-39).

PSQI was originally developed for measuring sleep quality among men and women. Acceptable psychometric properties have been reported for both genders (40). The PSQI has also been frequently used to test sex or gender differences in sleep quality (41). This questionnaire has been also successfully implemented for studying sleep quality among men (42) and women (43).

In this study, five socio-economic variables were the independent variables, sleep quality was the dependent variable, and gender was the moderator. Statistical analysis was conducted using the SPSS software package for Windows, version 13. Descriptive statistics are provided using means and Standard Deviations (SD). The associations between PSQI score and gender, education, and place of resistance were analyzed using independent sample t-test. Besides, the correlation between PSQI score and age was examined using Pearson correlation test (44). One-way ANOVA was used to evaluate the association between PSQI score and marital status as well as income (45). In addition, two-way ANOVA was used to evaluate the potential moderating effect of socio-economic data on sleep quality (39). As defined by Baron et al., moderator is a variable that affects the direction or / and the strength of the association between an independent and a dependent variable (29). P < 0.05 was considered as statistically significant.

## 3. Results

This study was conducted on 717 patients. Among the study participants, 65% (n = 467) were male and 35% (n = 250) were female. In addition, the mean (SD) age of the patients was 57.7 ± 11.7, with a range from 30 to 83 years. The clinical and socio-economic characteristics of the participants are presented in Table 1. 

**Table 1.** Socio-Economic and Clinical Characteristics of the 717 Patients with Coronary Artery Disease

Socio-Economic Characteristics	
**Gender**	
Male	467(65.1)
Female	250(34.9)
**Family Income**	
≤ $ 200	235(32.9)
$ 200 - 300	343(48.1)
$ 300 - 400	74(10.2)
≥ $ 400	65(8.9)
**Education**	
Not completed high school	476(66.5)
Completed high school	241(33.5)
**Marital status**	
Married	609(84.4)
Single	12(1.7)
Divorced	90(12.6)
Widowed	10(1.4)
**Residence**	
Urban	668(93.7)
Rural	49(6.3)
**Clinical characteristics Vessel disease**	
1	243(33.9)
2	222(30.9)
3	252(35.2)
**Cholesterol (mg / dL)**	
≥ 200	374(52.2)
< 200	343(47.8)
**Hypertension**	
Yes	563(78.5)
No	154(21.5)
**Diabetes mellitus**	
Yes	452(63)
No	265(37)
**Smoking**	
Current	302(42.1)
Past	136(19)
Never	279(38.9)

Independent sample t-test suggested that sleep quality was significantly poorer among the women compared to the men (9.72 ± 4.37 vs. 6.95 ± 4.21; P < 0.001). Moreover, ANOVA suggested that sleep quality was associated with education level, monthly income, and marital status, but not with living place (Table 2). 

**Table 2.** The Association between the Socio-Economic Characteristics and Sleep Quality among the 717 Patients with Coronary Artery Disease

	Mean	Std.	95% CI		P value
	Deviation for Mean				
			Lower Bound	Upper Bound	
**Gender**					< 0.001
Men	6.95	4.21	6.59	7.32	
Women	9.72	4.37	9.19	10.26	
**Education**					
Illiterate	8.52	4.37	7.91	9.12	0.002
1 - 5 years	8.14	4.55	7.51	8.77	
6 - 11 years	8.06	4.84	7.12	8.99	
**Completed**					
High school	7.63	4.49	6.92	8.34	
College or higher	6.35	3.70	5.59	7.10	
**Monthly income**					
≤ $ 200	8.98	4.64	8.40	9.56	< 0.001
$ 200 - 300	7.66	4.36	7.21	8.12	
$ 300 - 400	7.56	4.30	6.58	8.53	
≥ $ 400	5.90	3.65	5.00	6.79	
**Marital status**					
Married	7.76	4.42	7.42	8.10	0.002
Single	4.92	3.09	2.95	6.88	
Divorced	9.02	4.57	8.08	9.97	
Widowed	10.10	4.95	6.56	13.64	
**Urban residence**					
Urban	7.94	4.51	7.60	8.27	0.461
Rural	7.61	3.82	6.53	8.68	

Post hoc test indicated that within the education groups, those with college or higher had a better quality of sleep in comparison to the participants who were illiterate or those who had 1 - 5 years of education. Post hoc test also suggested that considering marital status, singles reported a better quality of sleep compared to those who were divorced or widowed. Furthermore, the patients with the lower income levels reported significantly poorer sleep quality compared to those with200 - 300 or more than 400 U.S. dollar incomes. Also, the participants with 200 - 300 U.S. dollar income had a lower sleep quality in comparison to those with the income of more than 400 U.S. dollars (Table 2). 

The results of two-way ANOVA revealed two significant interaction terms in this study. The first interaction was between gender and education level (P = 0.016) and the second one was between gender and monthly income (P = 0.019). Based on Baron and Kenny (49), educational level and income interacted with gender in influencing sleep quality.

**Figure 1.**
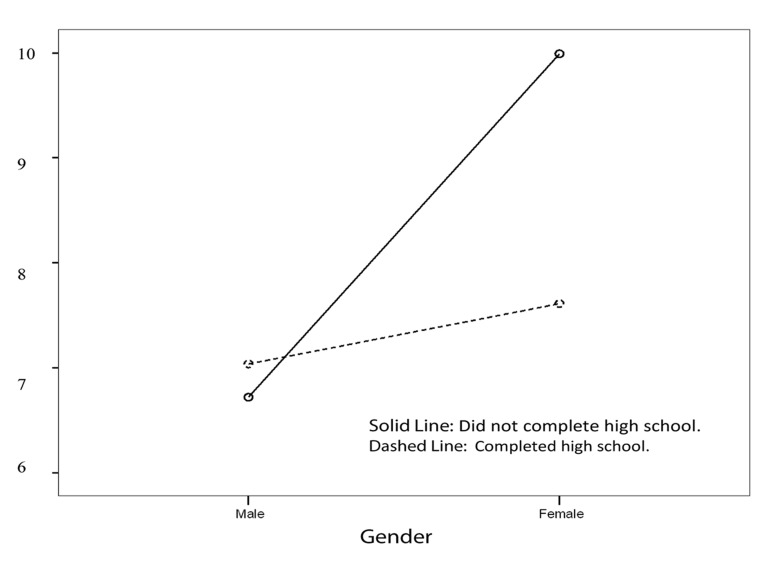
Interaction between Gender and Education on Sleep Quality (Pittsburgh Sleep Quality Index; PSQI Score)

Among the women with CAD, those who had not completed high school reported poorer sleep quality compared to those who had completed high school (10.0 ± 4.3 vs. 7.6 ± 5.0, P < 0.05). However, such a difference could not be found among the males (6.7 ± 4.2 vs. 7.0 ± 4.2, P > 0.05) (Figure 1). 

**Figure 2.**
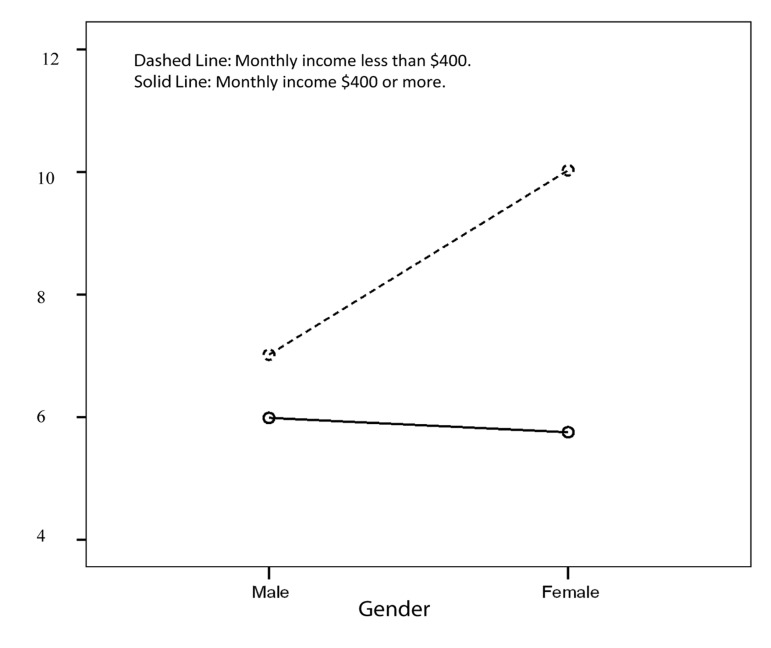
Interaction between Gender and Income on Sleep Quality (Pittsburgh Sleep Quality Index; PSQI Score)

Among the women with CAD, those with the monthly income of less than 400 U.S. dollars reported lower sleep quality in comparison to those with higher income levels (10.0 ± 4.3 vs. 5.7 ± 2.5, P < 0.05). Nonetheless, this difference could not be found among the male patients (7.0 ± 4.2 vs. 6.0 ± 3.8, P > 0.05) (Figure 2). 

Gender did not modify the effect of living place, occupation, and marital status on sleep quality.

## 4. Discussion

This study showed that gender interacted with the socio-economic characteristics, such as education and family income, on sleep quality among the patients with CAD. Our findings suggested that among the patients with CAD, women might be more vulnerable to the effects of low education and income on sleep quality. This implies that low education and income may contribute to gender differences in sleep quality among the patients with CAD. These results are critical because sleep quality is a prognostic factor among the patients with CAD.

The study findings showed that socio-economic status might be a mechanism behind gender differences in sleep quality among the CAD patients. This has been supported by the Theory of Gender and Power. According to this theory, sexual divisions of labor and power result in major gender differences in employment, income, and education. Such imbalances will result in subordination of the women in the society. As a result of these inequalities, life experiences differently influence the health and well-being of men and women (35).

In a study Edell-Gustafsson et al. (32) conducted on 135 patients with CAD (47 females and 88 males) to compare sleep quality in men and women with stable CAD, regression analysis showed that gender accounted for variance of sleep quality. In another study, one year after myocardial infarction, controlling for differences in age and co-morbidity, women reported significantly higher frequencies of psychological and psychosomatic complaints, including sleep disturbances (46). Despite some controversies exist regarding the influence of gender on sleep quality (5, 19, 27-30, 46, 47), there are several studies documenting poorer sleep quality among women compared to men (17-26). However, very few studies have evaluated this association among the patients with CAD.

Up to now, multiple mechanisms have been proposed to explain lower subjective and objective sleep quality among women. Menstrual cycle is an important factor affecting sleep quality in female gender. Previous research suggested that sleep pattern changed across the menstrual cycle (48-51) and by ovulation status (51-53). This may be due to the hormonal fluctuations and also changes in the plasma melatonin level across the menstrual cycle (25). Perceived stress, anxiety, and depression may be other factors that explain gender differences in sleep quality (25, 51-56). Insomnia is frequently a symptom of anxiety and depression which are both more prevalent among women. Thus, gender differences in the prevalence of insomnia might be due to the gender differences in the prevalence of anxiety and depression (54-56).

The present findings may contribute to our understanding about gender differences in sleep quality among the patients with CAD. Based on our findings, differential effects of socio-economic status may explain why a larger proportion of the women with CAD report poor sleep. Further studies should also consider how gender interacts with socio-economic status in shaping immune function, inflammation, and cardiovascular outcomes. One study has suggested that poor sleep quality predicted higher levels of circulating IL-6 and CRP among women, but not men (16). Another study has shown that poor subjective sleep quality was associated with inflammatory trajectories in women (12). Moreover, other studies have reported more strong longitudinal links between sleep and hypertension and cardiovascular events among women compared to men (57). As gender may interact with the effect of socio-economic status on sleep quality, future research in this area should include the socio-economic factors, as well.

To conclude, gender modifies the effect of income and education level on sleep quality among the patients with CAD. Thus, socio-economic factors may contribute to gender differences in sleep quality among these patients.
